# The Known Unknowns of the Immune Response to *Coccidioides*

**DOI:** 10.3390/jof7050377

**Published:** 2021-05-11

**Authors:** Rebecca A. Ward, George R. Thompson, Alexandra-Chloé Villani, Bo Li, Michael K. Mansour, Marcel Wuethrich, Jenny M. Tam, Bruce S. Klein, Jatin M. Vyas

**Affiliations:** 1 Department of Medicine, Division of Infectious Diseases, Massachusetts General Hospital, Boston, MA 02114, USA; raward@mgh.harvard.edu (R.A.W.); mkmansour@partners.org (M.K.M.); 2 Department of Internal Medicine, University of California Davis Medical Center, Sacramento, CA 96817, USA; grthompson@ucdavis.edu; 3 Center for Immunology and Inflammatory Diseases, Department of Medicine, Massachusetts General Hospital, Boston, MA 02114, USA; avillani@mgh.harvard.edu (A.-C.V.); bli28@mgh.harvard.edu (B.L.); 4 Broad Institute of MIT and Harvard, Cambridge, MA 02142, USA; 5 Harvard Medical School, Boston, MA 02115, USA; jenny.tam@wyss.harvard.edu; 6 Department of Pediatrics, School of Medicine and Public Health, University of Wisconsin-Madison, Madison, WI 53706, USA; mwuethri@wisc.edu (M.W.); bsklein@wisc.edu (B.S.K.); 7 Wyss Institute for Biologically Inspired Engineering, Harvard University, Boston, MA 02115, USA; 8 Department of Medicine, School of Medicine and Public Health, University of Wisconsin-Madison, Madison, WI 53706, USA; 9 Department of Medical Microbiology and Immunology, School of Medicine and Public Health, University of Wisconsin-Madison, Madison, WI 53706, USA

**Keywords:** coccidioidomycosis, innate immunity, adaptive immunity, respiratory epithelium, vaccine strategies, single-cell RNA sequencing, spatial transcriptomics

## Abstract

Coccidioidomycosis, otherwise known as Valley Fever, is caused by the dimorphic fungi *Coccidioides immitis* and *C. posadasii*. While most clinical cases present with self-limiting pulmonary infection, dissemination of *Coccidioides* spp. results in prolonged treatment and portends higher mortality rates. While the structure, genome, and niches for *Coccidioides* have provided some insight into the pathogenesis of disease, the underlying immunological mechanisms of clearance or inability to contain the infection in the lung are poorly understood. This review focuses on the known innate and adaptive immune responses to *Coccidioides* and highlights three important areas of uncertainty and potential approaches to address them. Closing these gaps in knowledge may enable new preventative and therapeutic strategies to be pursued.

## 1. Introduction

Coccidioidomycosis, also known as Valley Fever, is an invasive fungal infection caused by the dimorphic fungal pathogens *Coccidioides immitis* and *Coccidioides posadasii*. There are tens of thousands of reported cases of coccidioidomycosis annually, although this likely underestimates the true rate of occurrence due to underreporting. Contrary to many other pathogenic fungi that largely impact immunocompromised individuals, *Coccidioides* spp. cause a range of disease severity in their hosts regardless of their immune status (i.e., immunocompetent or immunocompromised). A potentially severe respiratory mycosis, the clinical spectrum of coccidioidomycosis ranges from mild pulmonary infection to disseminated, fatal mycosis. While numerous individuals are exposed to *Coccidioides*, only 40% develop symptomatic disease and reactivation may occur years after initial exposure [1]. Unfortunately, we do not fully understand the underlying mechanisms contributing to the diverse spectrum of disease in coccidioidomycosis. There is a critical need for individualized therapeutic strategies, but this requires greater mechanistic insight into the host-pathogen interactions that manifest a range of clinical phenotypes.

While *Coccidioides* spp. infect individuals with both intact and compromised immune systems, immunocompromised patients have an elevated risk of severe disease and dissemination. Indeed, individuals with HIV infection or hematologic malignancies, those undergoing solid organ or hematopoietic cell transplantation, or receiving immunosuppressive treatments (e.g., chemotherapy, glucocorticoids, and anti-tumor necrosis factor alpha (TNF-α) therapy) are at high risk of developing severe and/or complicated diseases [2]. Diabetes mellitus, rheumatoid arthritis, and pregnancy are also established risk factors for complicated coccidioidomycosis [3]. These high-risk groups highlight the critical role of the immune system in limiting *Coccidioides* infections. In addition to these co-morbid conditions, individuals with higher barriers to care are disproportionally impacted by worsened disease [3]. Unfortunately, we do not understand the molecular and cellular events leading to disseminated disease. 

Native to arid and semiarid conditions, *Coccidioides* spp. dwell in the southwestern United States, Mexico, and South America [4,5]. Cases in North-Eastern Utah and Washington demonstrate a significant expansion of historical boundaries, amplifying the number of humans and animals at risk of exposure [6,7,8]. These organisms have a biphasic life cycle, growing as arthroconidia and mycelium in the soil, and transforming into large spherules that eventually burst and spill hundreds of endospores once in a host [9,10]. This biphasic lifecycle and size difference between arthroconidia (2–4µm) and large spherules (80–100 µm) has slowed progress in the understanding of proper immune responses to *Coccidioides* spp. This review will examine the known factors and mechanisms of innate and adaptive immune responses to *Coccidioides*. Furthermore, we will highlight critical questions and potential solutions to further investigate host-pathogen responses in coccidioidomycosis. 

## 2. The Known Immune Responses to *Coccidioides* spp.

### 2.1. Innate Immunity

An intact immune system is critical for swift clearance of *Coccidioides* spp. pulmonary infections [11,12,13]. Many pathogens are effectively removed from the airways through mucociliary action. Neutrophils (PMNs), macrophages/monocytes, eosinophils, and natural killer (NK) cells are often early cellular responders to infection, while dendritic cells (DCs) complement and bridge the innate and adaptive responses. PMNs and monocytes are quick to respond to arthroconidia. Studies using murine models have shown conflicting results for the role of neutrophils depending on previous exposure to *Coccidioides*. Neutrophil-depleted C57BL/6 mice have similar susceptibility to wild-type mice; however, mice exposed to attenuated *Coccidioides* demonstrated a neutrophil-dependent protection against wild-type *Coccidioides* [14]. Phagocytosis by neutrophils and macrophages is a critical function in elimination of other fungal pathogens. However, the large size of mature *Coccidioides* spherules block ingestion by phagocytic cells, although smaller arthroconidia, immature spherules, and endospores can be engulfed [15]. Neutrophils rapidly respond to the release of endospores from mature spherules and inhibit endospore and spherule growth in vitro via oxidative burst [3,16]. Interestingly, neutrophils from patients with chronic coccidioidomycosis exhibited similar phagocytic abilities as neutrophils from healthy individuals [15]. It should be noted that chronic disease is associated with higher neutrophil infiltration [17]. While both neutrophils and monocytes readily phagocytose the arthroconidia, killing is not efficient and the organism may survive despite engulfment [18,19]. The ability of macrophages to neutralize *Coccidioides* endospores is reliant on the presence of interferon (IFN)-γ and TNFα, and in their absence, endospores halt phagolysosome formation and subsequent killing by macrophages [20,21,22].

Much of the current understanding of innate immune responses to *Coccidioides* spp. focuses on neutrophils and monocytes, but little is known about other critical innate immune cells (e.g., DCs, eosinophils, and NK cells). DCs can phagocytose fungal pathogens, secrete cytokines including IL-1β, IFN-γ and TNF-α and present antigens to naïve T cells, thus bridging the innate and adaptive immune response [23]. Recognition of spherules activates DCs leading to amplified CD40 and CD80/CD86 expression and subsequent activation of adaptive immune cells [24]. DCs are not one homogenous population. Little is known about the contribution of DC subtypes including conventional DCs (cDCs), plasmacytoid DCs (pDCs), and monocyte-derived DCs (moDCs) to host defense against *Coccidioides*. Eosinophils participate in the host defense by degranulation of secretory mediators, release of reactive oxygen species (ROS), and neutrophil extracellular traps (NET) formation. The precise role of eosinophils in *Coccidioides* infection remains poorly understood [25]. NK cells can kill fungal pathogens by secretion of perforin and granzyme and participate in the host defense against *Coccidioides* [26,27,28,29].

Fungal pathogens are recognized by pattern recognition receptors (PRRs) such as C-type lectin-like receptors (CLRs), Toll-like receptors (TLRs), NOD-like receptors (NLRs), and Rig-I-like receptors [30]. Similar to other pathogenic fungi, the outer wall of *Coccidioides* spp. is composed of chitin, β-glucan, and mannans [31]. In murine studies, Dectin-1, a CLR critical to antifungal immunity, recognizes and secretes pro-inflammatory cytokines in response to *Coccidioides* spherules, although the response is not uniform across different innate cells (i.e., macrophages and DCs) [12]. In the absence of Dectin-1, protective Th17 and Th1 cytokine responses and protective immune responses are drastically reduced. Similar to Dectin-1, loss of TLR2 or downstream MyD88/TRIF in mice resulted in diminished pro-inflammatory cytokine production in macrophages [12,23]. The CLRs Dectin-1, Dectin-2, and Mincle on monocytes and neutrophils interact with spherical outer wall glycoproteins (SOWgp) on arthroconidia and immature spherules [32,33]. Although these immune cells can recognize immature spherules, mature spherules evade detection through secretion of metalloproteinase 1 (Mep1) which degrades SOWgp [34]. Emerging data suggests a role for the NLR inflammasomes in innate responses to fungal pathogens, including *Candida albicans* and *Aspergillus* [35,36]. It is interesting to speculate that these cytosolic sensors may also play a role in *Coccidioides* spp. infection. Further investigation is warranted to dissect this potential pathway leading to the production of pro-inflammatory cytokines IL-1β and IL-18.

The secretome (the secretion of proteins to the external environment) is critical in maintaining cell–cell communication and recruitment of immune cells in response to pathogens. These secreted proteins include hormones, cytokines, chemokines, and growth factors. Cytokines including IFN-γ, IL-2, TNFα, IL-13, granulocyte-macrophage colony-stimulating factor (GM-CSF), and IL-1β are critical factors that enhance the innate immune response in primary pulmonary coccidioidomycosis [37]. IL-8 is a critical chemokine in recruitment of neutrophils to the site of infection. Indeed, mice lacking the IL-8 receptor 2 (IL-8R2) had reduced fungal burden compared to wild-type mice and skewed cytokine signature towards Th1 and Th17 phenotypes due to a higher expression of genes associated with lymphocyte activation [38]. It should be noted that while a reduced fungal burden was observed, no survival data was reported in this study. Further investigation into which cell types facilitate a protective cytokine signature is warranted. Cytokine signatures produced by innate immune cells are critical to protective responses by the adaptive immune system.

### 2.2. Adaptive Immunity

Guided by initial innate immune signatures, adaptive immune responses license the host to develop immunological memory and enhance protective immune responses. Granulomas restrain the growth and dissemination of many pathogens including *Mycobacterium tuberculosis* and *Histoplasma capsulatum* [39,40,41,42]. This unique tissue “niche” includes various cell types including epithelial cells, accessory cells, and T cells, the latter instrumental in microbial containment through production of Th1 type cytokine production (IFN-γ, TNFα, IL-12) [39]. Investigations into granuloma formation in *Coccidioides* infections will allow for better mechanistic understanding of what leads to chronic infection and disseminated disease. These results would provide insight into cell populations necessary for granuloma formation and may provide diagnostic and therapeutic avenues to pursue. Although there have been some studies investigating B cells in coccidioidomycosis, adaptive immunity to *Coccidioides* is primarily mediated through T cell responses [43,44,45,46]. Thus, we focus on T cell immunity in response to *Coccidioides* spp. in this review.

T cell differentiation enables targeted immune responses. As stated above, IFN-γ, TNFα, and IL-12 are all Th1 cytokines. Th17 differentiation is induced by IL-1β, IL-6, IL-23, and TGFβ. Th1 and Th17 cells are essential to adaptive responses to *Coccidioides* via mobilization of innate immune cells, further activation of adaptive immune cells, and production of anti-microbial molecules by endothelial cells [47]. Loss of either Th1 or Th17 responses lead to impaired fungal clearance and ultimately, worsened outcomes. CD4^+^ T-helper and CD8^+^ cytotoxic T cells contribute to adaptive responses against *Coccidioides* spp. via production of Th1 cytokines [48]. Loss of IL-1R results in reduction of Th17, but not Th1, T cells [14]. Patients with chronic pulmonary coccidioidomycosis have been noted to have differences in adaptive immunity. Elevated levels of regulatory T cells (Treg) were associated with persistent coccidioidomycosis in a pediatric population [17]. Furthermore, chronic infection was correlated with amplified expression of serum IL-10, a known Treg cytokine. Mice more resistant to *Coccidioides* infection (DBA/2) have lower number of Tregs and IL-10 production compared to susceptible mice (C57BL/6 and BALB/C), suggesting that Tregs may be detrimental in coccidioidomycosis [49,50,51].

Despite an active search for a vaccine, no candidate has received FDA approval [13,52,53,54]. A clinical trial using formaldehyde-killed spherules of *C. immitis* demonstrated no difference in coccidioidomycosis between the vaccine and placebo groups despite promising results in pre-clinical studies [55,56]. Attenuated strains targeting both chitinase 2 and 3 genes [57], CPS1 gene [58], the spherule outer wall glycoprotein (SOWgp) [33], or ammonia metabolism [59] have demonstrated reduced virulence and enhanced Th1 and/or Th17 responses upon challenge with wild-type *Coccidioides*. The multivalent recombinant coccidioidial Ag in the glucan-chitin particle (GCP-rCpa1) vaccine requires CARD-9-associated Decin-1 and Dectin-2 interactions for protective immunity [13]. CARD-9, Dectin-1, or Dectin-2 deficient mice failed to mount Th17 responses and lost vaccine-mediated protection. The live, attenuated Δ*cps1* strain is being advanced as a potential vaccine in dogs, yet the mechanism of protective immunity is not fully understood. In the Δ*cps1* vaccine strain, radial growth rate and spore morphology are similar to wild-type strains. Notably, the Δ*cps1* vaccine strain results in smaller spherules. Δ*cps1* induces a Th1 response in subcutaneously immunized mice [52]. DCs play a protective role in coccidioidomycosis, and DC-based vaccines (antigen2/PRA epitope and Δ*T*) trigger a potent cellular and humoral immune response [49,53].

Vaccine strategies with adjuvants enhance immune memory and are more effective. Addition of the human complement component C5a enhances Δ*T* vaccine-induced immunity via increased Th1 and Th17 responses, elevated effector cytokines, and higher IgG1 and IgG2 titers [60]. While these results are promising, more studies investigating adjuvant combinations with subunit vaccines or attenuated *Coccidioides* vaccine strains are warranted. Furthermore, most vaccine studies have included murine models only. Understanding correlates of protection by the human immune system and infected tissues will enable better vaccine strategies to be developed against *Coccidioides* spp.

## 3. Fundamental Questions of Host Immune Responses to *Coccidioides*

### 3.1. What Is the Role of Respiratory Epithelium in Host Defense against Coccidioides?

The respiratory epithelium is a collection of polarized cells that serve to partition the body’s internal milieu from the outside environment. Respiratory epithelium is the first point of contact between inhaled pathogens and the human host. Recent work has established that the respiratory epithelium is not a homogenous sheet of epithelial cells, but rather a complex mixture of different cell types with distinct transcriptional signatures and specialized functions [61,62,63]. Ciliated cells, goblet cells, ionocytes, club cells, tuft cells, pulmonary neuroendocrine cells (PNECs), and basal cells all comprise the respiratory epithelium and coordinate to maintain homeostasis. Clearance of pathogens via mucociliary action deter pathogens from disrupting the tissue environment [64,65]. Mucus secreted by goblet cells contain peptides with antimicrobial properties including β-defensins, lactoferrins, lysozyme, mucin, and surfactants [66]. Despite these anti-infective mechanisms, additional measures are necessary to prevent infection. 

Airway epithelium is an immunologically active tissue that may participate in pathogen phagocytosis [67,68] yet these data are hard to interpret as immortalized cell lines show significant capacity to phagocytose, a feature not seen in primary airway epithelium. Although resident alveolar macrophages and DCs play a role in initial host responses, it is increasingly evident that airway epithelial cells are essential in coordination of immune cell recruitment in the presence of inhaled pathogens in immunocompetent individuals [69]. Respiratory epithelial cells are equipped with PRRs (e.g., TLRs), which enable them to rapidly sense pathogens and recruit innate immune cells [70,71]. Furthermore, cytokine receptors on epithelial cells enable them to respond to immune cell signals. Production of cytokines that participate in inflammation (e.g., TNFα, GM-CSF, Gro-α [CXCL1], and IL-8) by the lung epithelium inform the immune system how to respond to invading microbes [65]. 

In addition to their ability to sense pathogens and secrete inflammatory mediators, respiratory epithelium has an intrinsic propensity for inflammatory memory [72]. In chronic rhinosinusitis patients, IL-4 and IL-13 responsive genes were upregulated in basal cells compared to healthy controls. Furthermore, these basal cells have epigenetic changes that contributed to persistent type 2 inflammatory milieu. While it is increasingly evident that respiratory epithelium is immunologically active, the sequence of events leading to the clearance of pathogens remains unknown; in other words, whether the stimulation and secretion of proinflammatory signals originates in the respiratory epithelial cells or immune cells. Little is known about the contribution of the respiratory epithelium in coccidioidomycosis. Many studies focus on the role of immune cells. Investigations using co-culture methods with primary airway epithelium and innate immune cells or in vivo modeling may provide greater insights into the contribution of respiratory epithelium in host defense against *Coccidioides* spp.

Human ex-vivo models have been limited by challenges in cell culture techniques. Nearly all published studies utilize immortalized cancer cell lines including A549 (adenocarcinomic human alveolar basal epithelial cells) and H292 (human lung mucoepidermoid carcinoma) cell lines [73,74,75] (Table 1). The chief advantages of these immortalized cell lines have been the substantial experience with response of these cell lines, amount of previously published data using these models, and the ease of growth and the ability to grow these cells in bulk. The limitation of using these human cell lines is their monomorphic oncogenic nature that does not recapitulate fully differentiated, pseudostratified characteristics of primary human airway epithelium. For example, these cell lines lack ionocytes, a rare epithelial cell type that is the main source of the cystic fibrosis transmembrane conductance regulator (CFTR), an important component of cystic fibrosis (CF) [61,63]. Furthermore, cell–cell communication between multiple epithelial cell subtypes is lost in these cell lines.

Recently, a novel method for extended culturing (>10 passages) of patient-derived pulmonary hAECs has been developed [62]. These cells are isolated from patient sputum, bronchoalveolar lavage (BAL), or surgical explants (Table 1). Basal cells isolated from these clinical samples are differentiated into an air–liquid interface (ALI) culture system to complete airway epithelium. These cells recapitulate the true complexity of airway epithelium with common cell types (ciliated cells, goblet cells, club cells, basal cells) and rare cell types (tuft cells, PNECs, and ionocytes) [61,63]. Unlike a monolayer of H292 and A549 cells, these differentiated cells form a pseudostratified layer and demonstrate coordinated cilia beating at ALI [76]. This model has been used to culture differentiated primary hAECs from healthy subjects and patients with CF, chronic obstructive pulmonary disease (COPD), and asthma [76,77]. It remains formally possible that during differentiation of basal cells, these hAECs will no longer resemble the phenotypic and functional profiles of the host epithelial cells because they have been differentiated ex vivo from the lung environment. Nevertheless, this model is more advantageous than induced pluripotent stem cells since these basal cells retain imprinted epigenetics inherent to the host [61,78]. Indeed, hAECs from patients with COPD and asthma demonstrate important differences in SMAD signaling that correlate to goblet hyperplasia [77]. This model can also be used from murine cells, which will enable better understanding of species differences [79].

Culturing of primary hAECs allows researchers to interrogate human responses to clinically relevant pathogens. This model was used to demonstrate the neutrophil-derived cytosolic phospholipase A2 α isoform (cPLA2α) is critical in neutrophil migration in responses to the bacteria Pseudomonas aeruginosa [80]. In a fungal model that utilized primary hAECs, melanin shields epitopes in A. fumigatus to blunt neutrophil recruitment [76]. Expansion of hAEC infection modeling to *Coccidioides* may provide important insights into airway-mediated host defense. Furthermore, the ability to isolate basal cells from different patient populations (i.e., chronic or disseminate coccidioidomycosis) will enable dissection of underlying differences contributing to dissemination. Although primary in vitro models can provide insight into human cells, they lack the complexity (e.g., complement, immune cells, endothelial cells, etc.) seen in vivo. Thus, animal models are essential to validate in vitro results and extend our understanding of fungal infections. 

Transgenic mice and immunosuppressive models have been leveraged to dissect the role of respiratory epithelium in fungal infections (Table 2). IκB kinase (IKK)^∆LEC^ mice constitutively lack NFκB signaling in lung epithelium broadly. In *H. capsulatum* infection, impaired NFκB signaling in lung epithelium impairs control of infection, survival and priming of antigen specific CD4^+^ T cells. There are multiple models to investigate the contribution of pulmonary club cells including IκB-α dominant inhibitor transactivated (DNTA) transgenic mice lacking NFκB signaling only in club cells upon doxycycline treatment [81]. Club cells regulate resistance to the fungal dimorph *Blastomyces dermatitidis* by elaborating products such as CCL2, CCL20 and IL-1 to assemble myeloid and lymphoid cells that restrain the fungus [82]. Club cells also regulate allergic inflammation in response to inhaled *Aspergillus* protease allergens [83]. Mucin production by epithelial cells is one of the first lines of defense against pathogens. Recently, a *Muc5b* haplo-insufficient model, which targets the mucin MUC5b, demonstrated severe respiratory distress in some mice [84]. This group additionally characterized a conditional *Muc5b* knockout in pulmonary club cells using the SCGB1A promoter. These models have not been used to investigate signaling pathways in fungal infections. Goblet cell hyperreactivity and subsequent overproduction of MUC5AC has been associated with pathological functionality in pulmonary disease [85]. Thus, overexpression of *Muc5ac* in the lungs may provide insights into pathogen clearance in the setting of hyperreactivity [86]. *Ascl1* conditional knockout mice have no PNECs. This model was utilized to demonstrate that PNEC regulate innate lymphoid cell responses to inhaled allergens [87]. Limited studies have investigated the contribution of ionocytes to pulmonary disease. The first study to characterize this subtype used Foxi1KO to deplete ionocytes in vivo [61]. These transgenic mouse models provide the resources for targeted studies of the host airway responses to fungal pathogens. While these existing models will be useful, further investigations into patient-specific transcriptional differences are necessary and complementary to animal models.

### 3.2. Are There Correlates of Infection Outcomes and Transcriptional Changes in Respiratory Epithelium and Immune Cells That Define a Protective Immune Response?

Dissemination of *Coccidioides* spp. is a dreaded outcome in patients [88]. Although some risk factors have been associated with disease severity and dissemination in coccidioidomycosis, little is known about underlying factors contributing to disease severity. Mutations in IFN-γ or IL-12 receptor have been found in some patients with disseminated coccidioidomycosis, suggesting these pathways are critical to clearance of the infection [89,90]. Additionally, severe disseminated disease has been associated with a STAT3 mutation in some, but not all coccidioidomycosis patients [91]. Further investigations into polymorphisms and transcriptional changes in coccidioidomycosis are warranted.

Advances in transcriptional technologies and computational analyses enables the investigation into disease states and systems immunology, providing critical insights into numerous diseases including cancer, gastrointestinal diseases, and infections [92,93,94]. The Human Cell Atlas and similar consortiums are charting cell atlases of multiple complex tissues to understand health and disease [95,96]. These technologies include bulk RNA sequencing, single cell RNA sequencing (scRNA-seq), single nucleus RNA sequencing (snRNA-seq), and paired sequencing. Of these approaches, bulk RNA sequence is the least powerful, since it primarily reflects the average gene expression across all cells [97]. The contribution of rare cell populations, such as ionocytes in respiratory epithelium or plasmacytoid DCs, may be obscured by bulk analyses.

The emergence of single-cell profiling allows for unbiased methodology by utilizing next-generation sequencing. Sequencing of blood samples and dissociated cells from tissues of both human and mice have undergone scRNA-seq. This field is rapidly advancing, and thus numerous approaches for scRNA-seq are available (e.g., plate-based, bead-based, and combinatorial index-based methods) [98,99,100,101,102,103,104]. These methods differ by how they tag transcripts and generate libraries. Systematic comparison of seven scRNA-seq methods concluded that the high-throughput 10× Chromium had the strongest consistent performance [105]. Additionally, the low-throughput plate-based methods investigated, Smart-seq2 and CEL-Seq2, were better for higher sensitivity compared to high-throughput approaches. Factors such as cost effectiveness, run time, and number of cells should be considered when determining which method to utilize. Previous reviews on scRNA-seq approaches discuss these methodologies in greater detail [106,107].

Cell hashing may be used in conjunction with scRNA-seq when there are several samples to be analyzed in parallel by individually barcoding each sample and then running sequencing steps with pooled samples [108]. The power of these transcription techniques is amplified by enabling multimodal single-cell phenotypes with paired analyses for T cell receptors (TCR) or surface protein measurements using Cellular Indexing of Transcriptomes and Epitopes by Sequencing (CITE-seq) [102,109]. These paired protocols enable multiple readouts from one data set. Novel computational platforms are needed to enable the analysis of large-scale, multimodal single-cell genomics datasets. Cumulus, the first comprehensive cloud-based scRNA-seq data analysis platform, provides a quicker, more cost-effective way to address this analysis need [110]. Cumulus supports analysis from a variety of input modalities, including droplet-based [101,102] (3′ or 5′ ends, with unique molecular identifiers, UMIs) and plate-based [98] (full-length, no UMI) sc/snRNA-seq, CITE-seq, cell hashing or nucleus hashing experiments, which can be demultiplexed using a novel probabilistic algorithm [108,109,111]. Perturb-seq methods are used for pooled CRISPR screens with scRNA-seq readout [112,113,114,115,116].

These technologies have been leveraged to better understand immune responses and lung cell heterogeneity. Novel cell types, including subtypes of DCs, monocytes, innate lymphoid cells (ILCs), and ionocytes, have been discovered by scRNA-seq [61,117,118]. These discoveries are critical to understand the influence of rare cell types in the immune system and impacted organs in diseased states. Indeed, profiling of Th17 cells in experimental autoimmune encephalomyelitis demonstrated that rather than one homogenous population, these Th17 were highly heterogenous [119]. RNA-seq has been leveraged to understand infectious diseases. Recently, a pre-print highlighted observable differences is disease severity in SARS-CoV-2 infection [93]. Furthermore, a genome-wide association study in peripheral blood mononuclear cells from candidemia patients identified LY86 as an important factor in host defense against *Candida* [120]. In validation studies, LY86 deficiency reduced MCP-1-mediated monocyte migration in response to *Candida* infection in vitro. Transcriptional studies of the immune system and lung in coccidioidomycosis can measure how pertinent cells change and interact over the course of disease, pinpoint genetic variants, and highlight critical cell types and response required for a protective immunity. Primary human samples or in vitro models undergo dissociation protocols for scRNA-seq. A hallmark of coccidioidomycosis is the formation of granulomas. Understanding transcriptional changes in a granuloma within a spatial framework may provide greater insights into limited versus disseminate coccidioidomycosis.

### 3.3. What Can We Learn from Banked Tissues with Granulomas from Coccidioidomycosis Patients?

Granulomas are a method of immune defense that is thought to limit the spread of difficult to eradicate pathogens, Formation of granulomas are observed in many infections, including M. tuberculosis and fungal infections. Unfortunately, the molecular mechanisms leading to pathogen eradication in coccidioidomycosis are poorly understood. Patients with limited disease typically have granulomas that contain immune cells, epithelioid cells, and *Coccidioides*. Insights into M. tuberculosis, another pathogen that induces granuloma formation, has revealed that IL-18 from lung epithelium promotes granuloma formation [121]. Additionally, increased MMP leads to enhanced recruitment of macrophages and earlier granuloma formation [122,123]. These observations provide credence to the hypothesis that the lung epithelium controls granuloma formation in coccidioidomycosis patients. Within the endemic region, there are occasions when a patient has a tumor concerning for malignancy in the lung and will get an excisional biopsy [124]. Instead of discovering a malignancy (lung primary or metastatic cancer), the pathological analysis reveals a granulomatous lesion containing *Coccidioides*. In other cases, granulomas in extra-pulmonary sites (e.g., the heart, brain, skin) are identified [125]. These formalin-fixed, paraffin-embedded (FFPE) preserved tissues should be interrogated to better understand granuloma formation in humans.

Basic staining methods are routine in tissue samples from coccidioidomycosis patients. Hematoxylin and eosin (H&E) staining, which has been used with tissues from coccidioidomycosis patients [125,126], is a routine histopathological method. Hematoxylin stains nucleic acids a deep purple, while eosin stains extracellular matrix and cytoplasm pink. H&E can define morphologic changes and can provide insight into infiltrating immune cells and tissue damage. Periodic acid-Schiff stain (PAS), Steiner silver stain, and Grocott’s methanamine silver (GMS) stain can be used to visualize *Coccidioides* [125]. Immunohistochemical and immunofluorescent staining may be leveraged to investigate targeted protein expression. While these methods offer information on the tissue, including immune cell infiltration, morphological form of *Coccidioides*, and tissue damage, basic histopathology methodology does not provide a window into relevant pathways activated during infection.

Advances in sequencing technologies enable targeted spatial transcriptomics of fixed tissues. The spatial layout of cells provides insight into interactions of biological networks in infected regions. Similar to scRNA-seq, multiple approaches to spatially resolved transcriptomics have been developed. Methodologies include in situ sequencing technologies, in situ hybridization technologies, in situ capturing technologies, micro-dissected gene expression technologies, and in silico reconstruction technologies. Since many coccidioidomycosis tissues are FFPE, we will focus on technologies that been used FFPE. Most spatial transcriptomic approaches require fresh-frozen tissue due to reduction in RNA quality that occurs in the fixation process of FFPE tissues. Further description of other spatially resolved transcriptomics technologies have been reviewed previously [127].

In situ hybridization technologies enable visualization of transcripts within the sample tissue rather by hybridizing labeled probes to transcripts of interest. FFPE tissues can leverage these technologies using a fluorescent probe in single-molecular RNA fluorescence in situ hybridization (smFISH) or “Z-probes” in RNAscope [128,129,130,131]. While both these methods have subcellular resolution, the throughput is low due to imaging and technical constraints. In situ sequencing using a single stranded DNA padlock probes allows for higher targets (up to 100) and amplified targets by rolling-circle amplification (RCA) [132]. Since RCA results in micrometer-sized products, this method has sub-cellular resolution.

Fluorescent in situ sequencing (FISSEQ) enables genome-wide profiling of gene expression in cells and tissues [133]. This is the only spatially resolved transcriptional approach validated in FFPE that is untargeted. However, detection efficiency and sensitivity were low and thus, could be problematic in tissue when detecting low levels of RNA targets in certain disease states. Recently, the group that developed FISSEQ optimized this approach by directly targeting mRNA, resulting in higher efficiency and lower background, thereby increasing signal-to-noise [134], used padlock probes specifically designed for target mRNA, amplified the circularized proves, and sequenced these barcodes over multiple rounds. BOLORAMIS allows for visualization of transcripts and uncovers the spatial relationship between cells and transcripts via gene-gene proximity and single-cell clustering analyses.

Lastly, Nanostring GeoMx allows for investigators to target their region of interest (ROIs; 10-600 µm) with a high level of automation [135]. Unfortunately, for smaller ROIs, there is low sensitivity. Spatially resolved technologies are continuing to be fine-tuned and if applied to coccidioidomycosis granuloma samples, may provide substantial insight into the mechanisms of containment and dissemination of *Coccidioides* spp.

## 4. Conclusions

**Figure 1:**
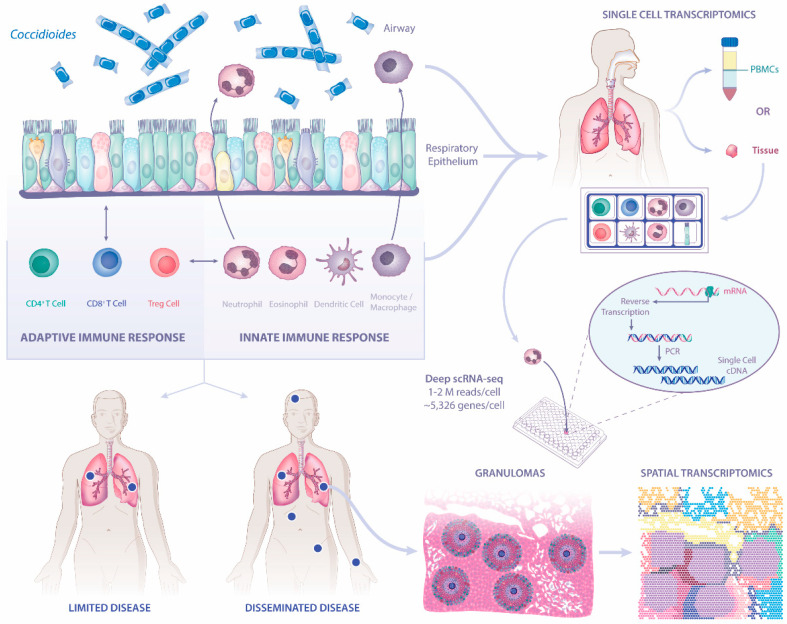
Future pathways of investigation to dissect disease severity in coccidioidomycosis. Immune responses by respiratory epithelial cells, the first point of contact, as well as innate and adaptive immune cells to *Coccidioides* will enable better understanding of protective immunity. Novel transcriptional technologies, including single-cell RNA sequencing [scRNA-seq] and spatial transcriptomics, can be leveraged using dissociated cells or tissues.

Coccidioidomycosis impacts both immunocompromised and immunocompetent individuals. Although we have some understanding of the mechanisms of action for innate and adaptive immunity in response to *Coccidioides* spp., the underlying cellular and molecular mechanism that account for differences in disease severity remains a critical gap in knowledge. Expanding investigations to include the role of the respiratory epithelium, the first line of defense to inhaled pathogens, in tandem with immune cells is essential (Figure 1). Furthermore, leveraging unbiased scRNA-seq technologies and spatially resolved transcriptomics in clinical samples from coccidioidomycosis patients may demonstrate critical pathways leading to dissemination. Together, these approaches will provide insights into novel therapeutic and preventative strategies against coccidioidomycosis.

## Figures and Tables

**Table 1:** Selected human respiratory epithelial cell in vitro cell models.

Model Name	Source	Advantages	Disadvantages
A549	Adenocarcinomic human alveolar basal epithelial cells	Ability to passage longer	Monolayer Lacks cell complexity Lack beating cilia Lack of supporting cells
NCI-H292	Human lung muco-epidermoid carcinoma	Ability to passage longer	Monolayer Express multiple markers of squamous differentiation Lacks cell complexity Lack beating cilia Lack of supporting cells
hAECs	Patient sputum, BAL, surgical explants	Recapitulates in vivo complexity Pseudostratified Beating cilia Models different disease states Retains host epigenetics	May lack functional profile of host Lack of supporting cells

**Table 2:** Transgenic murine models targeting respiratory epithelial cell subtypes.

Target Cell	Murine Line	Mutation	Models Investigated Using Line
Respiratory epithelium	IKK^∆LEC^	Lack NFκB signaling in lung epithelium	*B. dermatitidis, H. capsulatum, C. neoformans*
Club cells	DNTA	Lack NFκB signaling in club cells	*B. dermatitidis, H. capsulatum*
	Muc5b^Lung KO^	Conditional *Muc5b* KO using SCGB1A promoter	Characterized
Goblet cells	Muc5ac-Tg	Overexpression of *Muc5ac* mRNA	Influenza
PNECs	Ascl1CKO	Deficient in *Ascl1* in PNEC progenitors	OVA asthma model
Ionocyte	Foxi1KO	Deficient in *Foxi1*	CF

## References

[1] Thompson G.R. (2011). Pulmonary coccidioidomycosis. Semin. Respir. Crit. Care Med..

[2] Blair J.E., Ampel N.M., Hoover S.E. (2019). Coccidioidomycosis in selected immunosuppressed hosts. Med. Mycol..

[3] Castro-Lopez N., Hung C.Y. (2017). Immune response to coccidioidomycosis and the development of a vaccine. Microorganisms.

[4] Weaver E.A., Kolivras K.N. (2018). Investigating the relationship between climate and valley fever (coccidioidomycosis). Ecohealth.

[5] Ampel N.M. (2020). Coccidioidomycosis: Changing concepts and knowledge gaps. J. Fungi.

[6] McCotter O.Z., Benedict K., Engelthaler D.M., Komatsu K., Lucas K.D., Mohle-Boetani J.C., Oltean H., Vugia D., Chiller T.M., Sondermeyer Cooksey G.L. (2019). Update on the epidemiology of coccidioidomycosis in the United States. Med. Mycol..

[7] Oltean H.N., Springer M., Bowers J.R., Barnes R., Reid G., Valentine M., Engelthaler D.M., Toda M., McCotter O.Z. (2020). Suspected locally acquired coccidioidomycosis in human, Spokane, Washington, USA. Emerg. Infect. Dis..

[8] Johnson S.M., Carlson E.L., Fisher F.S., Pappagianis D. (2014). Demonstration of *Coccidioides immitis* and *Coccidioides posadasii* DNA in soil samples collected from Dinosaur National Monument, Utah. Med. Mycol..

[9] Cole G.T., Sun S.H. (1985). Arthroconidium-spherule-endospore transformation in *Coccidioides immitis*. Fungal Dimorphism—With Emphasis on Fungi Pathogenic for Humans, Szaniszlo, P.J., Harris, J.L., Eds..

[10] Kollath D.R., Miller K.J., Barker B.M. (2019). The mysterious desert dwellers: *Coccidioides immitis* and *Coccidioides posadasii*, causative fungal agents of coccidioidomycosis. Virulence.

[11] Hung C.Y., Hsu A.P., Holland S.M., Fierer J. (2019). A review of innate and adaptive immunity to coccidioidomycosis. Med. Mycol..

[12] Viriyakosol S., Fierer J., Brown G.D., Kirkland T.N. (2005). Innate immunity to the pathogenic fungus *Coccidioides posadasii* is dependent on Toll-like receptor 2 and Dectin-1. Infect. Immun..

[13] Campuzano A., Zhang H., Ostroff G.R., Dos Santos Dias L., Wüthrich M., Klein B.S., Yu J.J., Lara H.H., Lopez-Ribot J.L., Hung C.Y. (2020). CARD9-associated Dectin-1 and Dectin-2 are required for protective immunity of a multivalent vaccine against *Coccidioides posadasii* infection. J. Immunol..

[14] Hung C.Y., Jiménez-Alzate Mdel P., Gonzalez A., Wüthrich M., Klein B.S., Cole G.T. (2014). Interleukin-1 receptor but not Toll-like receptor 2 is essential for MyD88-dependent Th17 immunity to *Coccidioides* infection. Infect. Immun..

[15] Lee C.Y., Thompson G.R., Hastey C.J., Hodge G.C., Lunetta J.M., Pappagianis D., Heinrich V. (2015). *Coccidioides* endospores and spherules draw strong chemotactic, adhesive, and phagocytic responses by individual human neutrophils. PLoS ONE.

[16] Hung C.Y., Castro-Lopez N., Cole G.T. (2016). Card9- and MyD88-mediated gamma interferon and nitric oxide production is essential for resistance to subcutaneous *Coccidioides posadasii* infection. Infect. Immun..

[17] Davini D., Naeem F., Phong A., Al-Kuhlani M., Valentine K.M., McCarty J., Ojcius D.M., Gravano D.M., Hoyer K.K. (2018). Elevated regulatory T cells at diagnosis of Coccidioides infection associates with chronicity in pediatric patients. J. Allergy Clin. Immunol..

[18] Galgiani J.N. (1986). Inhibition of different phases of Coccidioides immitis by human neutrophils or hydrogen peroxide. J. Infect. Dis..

[19] Borchers A.T., Gershwin M.E. (2010). The immune response in coccidioidomycosis. Autoimmun. Rev..

[20] Beaman L. (1991). Effects of recombinant gamma interferon and tumor necrosis factor on in vitro interactions of human mononuclear phagocytes with Coccidioides immitis. Infect. Immun..

[21] Beaman L., Benjamini E., Pappagianis D. (1981). Role of lymphocytes in macrophage-induced killing of Coccidioides immitis in vitro. Infect. Immun..

[22] Beaman L., Benjamini E., Pappagianis D. (1983). Activation of macrophages by lymphokines: Enhancement of phagosome-lysosome fusion and killing of *Coccidioides immitis*. Infect. Immun..

[23] Viriyakosol S., Jimenez Mdel P., Gurney M.A., Ashbaugh M.E., Fierer J. (2013). Dectin-1 is required for resistance to coccidioidomycosis in mice. mBio.

[24] Dionne S.O., Podany A.B., Ruiz Y.W., Ampel N.M., Galgiani J.N., Lake D.F. (2006). Spherules derived from *Coccidioides posadasii* promote human dendritic cell maturation and activation. Infect. Immun..

[25] Simon H.U., Yousefi S., Germic N., Arnold I.C., Haczku A., Karaulov A.V., Simon D., Rosenberg H.F. (2020). The cellular functions of eosinophils: Collegium Internationale Allergologicum (CIA) update 2020. Int. Arch. Allergy Immunol..

[26] Petkus A.F., Baum L.L. (1987). Natural killer cell inhibition of young spherules and endospores of *Coccidioides immitis*. J. Immunol..

[27] Donovan F.M., Shubitz L., Powell D., Orbach M., Frelinger J., Galgiani J.N. (2019). Early events in coccidioidomycosis. Clin. Microbiol. Rev..

[28] Schmidt S., Tramsen L., Lehrnbecher T. (2017). Natural killer cells in antifungal immunity. Front. Immunol..

[29] Schmidt S., Zimmermann S.Y., Tramsen L., Koehl U., Lehrnbecher T. (2013). Natural killer cells and antifungal host response. Clin. Vaccine Immunol..

[30] Ward R.A., Vyas J.M. (2020). The first line of defense: Effector pathways of anti-fungal innate immunity. Curr. Opin. Microbiol..

[31] Cole G.T., Hung C.Y. (2001). The parasitic cell wall of *Coccidioides immitis*. Med. Mycol..

[32] Nguyen C., Barker B.M., Hoover S., Nix D.E., Ampel N.M., Frelinger J.A., Orbach M.J., Galgiani J.N. (2013). Recent advances in our understanding of the environmental, epidemiological, immunological, and clinical dimensions of coccidioidomycosis. Clin. Microbiol. Rev..

[33] Hung C.Y., Yu J.J., Seshan K.R., Reichard U., Cole G.T. (2002). A parasitic phase-specific adhesin of *Coccidioides immitis* contributes to the virulence of this respiratory fungal pathogen. Infect. Immun..

[34] Hung C.Y., Seshan K.R., Yu J.J., Schaller R., Xue J., Basrur V., Gardner M.J., Cole G.T. (2005). A metalloproteinase of *Coccidioides posadasii* contributes to evasion of host detection. Infect. Immun..

[35] Camilli G., Griffiths J.S., Ho J., Richardson J.P., Naglik J.R. (2020). Some like it hot: *Candida* activation of inflammasomes. PLoS Pathog..

[36] Briard B., Fontaine T., Samir P., Place D.E., Muszkieta L., Malireddi R.K.S., Karki R., Christgen S., Bomme P., Vogel P. (2020). Galactosaminogalactan activates the inflammasome to provide host protection. Nature.

[37] Ampel N.M., Robey I., Nguyen C.T., Roller B., August J., Knox K.S., Pappagianis D. (2018). Ex vivo cytokine release, determined by a multiplex cytokine assay, in response to coccidioidal antigen stimulation of whole blood among subjects with recently diagnosed primary pulmonary coccidioidomycosis. mSphere.

[38] Carlin A.F., Viriyakosol S., Okamoto S., Walls L., Fierer J. (2020). Interleukin-8 Receptor 2 (IL-8R2)-Deficient Mice Are More Resistant to Pulmonary Coccidioidomycosis than Control Mice. Infect. Immun..

[39] Hogan L.H., Macvilay K., Barger B., Co D., Malkovska I., Fennelly G., Sandor M. (2001). Mycobacterium bovis strain bacillus Calmette-Guerin-induced liver granulomas contain a diverse TCR repertoire, but a monoclonal T cell population is sufficient for protective granuloma formation. J. Immunol..

[40] Co D.O., Hogan L.H., Il-Kim S., Sandor M. (2004). T cell contributions to the different phases of granuloma formation. Immunol. Lett..

[41] Co D.O., Hogan L.H., Kim S.I., Sandor M. (2004). Mycobacterial granulomas: Keys to a long-lasting host-pathogen relationship. Clin. Immunol..

[42] Heninger E., Hogan L.H., Karman J., Macvilay S., Hill B., Woods J.P., Sandor M. (2006). Characterization of the Histoplasma capsulatum-induced granuloma. J. Immunol..

[43] Magee D.M., Friedberg R.L., Woitaske M.D., Johnston S.A., Cox R.A. (2005). Role of B cells in vaccine-induced immunity against coccidioidomycosis. Infect. Immun..

[44] Beaman L.V., Pappagianis D., Benjamini E. (1979). Mechanisms of resistance to infection with *Coccidioides immitis* in mice. Infect. Immun..

[45] Zhu Y., Tryon V., Magee D.M., Cox R.A. (1997). Identification of a *Coccidioides immitis* antigen 2 domain that expresses B-cell-reactive epitopes. Infect. Immun..

[46] Hung C.Y., Ampel N.M., Christian L., Seshan K.R., Cole G.T. (2000). A major cell surface antigen of *Coccidioides immitis* which elicits both humoral and cellular immune responses. Infect. Immun..

[47] Hung C.Y., Wozniak K.L., Cole G.T. (2016). Flow cytometric analysis of protective T-cell response against pulmonary *Coccidioides* infection. Methods Mol. Biol..

[48] Nesbit L., Johnson S.M., Pappagianis D., Ampel N.M. (2010). Polyfunctional T lymphocytes are in the peripheral blood of donors naturally immune to coccidioidomycosis and are not induced by dendritic cells. Infect. Immun..

[49] Hung C.Y., Castro-Lopez N., Cole G.T. (2014). Vaccinated C57BL/6 mice develop protective and memory T cell responses to *Coccidioides posadasii* infection in the absence of interleukin-10. Infect. Immun..

[50] Del Pilar Jiménez A.M., Viriyakosol S., Walls L., Datta S.K., Kirkland T., Heinsbroek S.E., Brown G., Fierer J. (2008). Susceptibility to *Coccidioides* species in C57BL/6 mice is associated with expression of a truncated splice variant of Dectin-1 (Clec7a). Genes Immun..

[51] Fierer J., Walls L., Eckmann L., Yamamoto T., Kirkland T.N. (1998). Importance of interleukin-10 in genetic susceptibility of mice to *Coccidioides immitis*. Infect. Immun..

[52] Shubitz L.F., Powell D.A., Trinh H.T., Lewis M.L., Orbach M.J., Frelinger J.A., Galgiani J.N. (2018). Viable spores of *Coccidioides posadasii Deltacps1* are required for vaccination and provide long lasting immunity. Vaccine.

[53] Awasthi S., Vilekar P., Conkleton A., Rahman N. (2019). Dendritic cell-based immunization induces *Coccidioides* Ag2/PRA-specific immune response. Vaccine.

[54] Hung C.Y., Zhang H., Castro-Lopez N., Ostroff G.R., Khoshlenar P., Abraham A., Cole G.T., Negron A., Forsthuber T., Peng T. (2018). Glucan-chitin particles enhance Th17 response and improve protective efficacy of a multivalent antigen (rCpa1) against pulmonary *Coccidioides posadasii* infection. Infect. Immun..

[55] Pappagianis D. (1993). Evaluation of the protective efficacy of the killed Coccidioides immitis spherule vaccine in humans. The Valley Fever Vaccine Study Group. Am. Rev. Respir. Dis..

[56] Pappagianis D., Hector R., Levine H.B., Collins M.S. (1979). Immunization of mice against coccidioidomycosis with a subcellular vaccine. Infect. Immun..

[57] Xue J., Chen X., Selby D., Hung C.Y., Yu J.J., Cole G.T. (2009). A genetically engineered live attenuated vaccine of *Coccidioides posadasii* protects BALB/c mice against coccidioidomycosis. Infect. Immun..

[58] Narra H.P., Shubitz L.F., Mandel M.A., Trinh H.T., Griffin K., Buntzman A.S., Frelinger J.A., Galgiani J.N., Orbach M.J. (2016). A *Coccidioides posadasii* CPS1 deletion mutant is avirulent and protects mice from lethal infection. Infect. Immun..

[59] Wise H.Z., Hung C.Y., Whiston E., Taylor J.W., Cole G.T. (2013). Extracellular ammonia at sites of pulmonary infection with *Coccidioides posadasii* contributes to severity of the respiratory disease. Microb. Pathog..

[60] Hung C.Y., Hurtgen B.J., Bellecourt M., Sanderson S.D., Morgan E.L., Cole G.T. (2012). An agonist of human complement fragment C5a enhances vaccine immunity against *Coccidioides* infection. Vaccine.

[61] Montoro D.T., Haber A.L., Biton M., Vinarsky V., Lin B., Birket S.E., Yuan F., Chen S., Leung H.M., Villoria J. (2018). A revised airway epithelial hierarchy includes CFTR-expressing ionocytes. Nature.

[62] Mou H., Vinarsky V., Tata P.R., Brazauskas K., Choi S.H., Crooke A.K., Zhang B., Solomon G.M., Turner B., Bihler H. (2016). Dual SMAD signaling inhibition enables long-term expansion of diverse epithelial basal cells. Cell Stem Cell.

[63] Plasschaert L.W., Zilionis R., Choo-Wing R., Savova V., Knehr J., Roma G., Klein A.M., Jaffe A.B. (2018). A single-cell atlas of the airway epithelium reveals the CFTR-rich pulmonary ionocyte. Nature.

[64] Caffrey-Carr A.K., Kowalski C.H., Beattie S.R., Blaseg N.A., Upshaw C.R., Thammahong A., Lust H.E., Tang Y.W., Hohl T.M., Cramer R.A. (2017). Interleukin 1alpha is critical for resistance against highly virulent *Aspergillus fumigatus* isolates. Infect. Immun..

[65] Espinosa V., Rivera A. (2016). First line of defense: Innate cell-mediated control of pulmonary aspergillosis. Front. Microbiol..

[66] Hallstrand T.S., Hackett T.L., Altemeier W.A., Matute-Bello G., Hansbro P.M., Knight D.A. (2014). Airway epithelial regulation of pulmonary immune homeostasis and inflammation. Clin. Immunol..

[67] Croft C.A., Culibrk L., Moore M.M., Tebbutt S.J. (2016). Interactions of *Aspergillus fumigatus* conidia with airway epithelial cells: A critical review. Front. Microbiol..

[68] Feldman M.B., Vyas J.M., Mansour M.K. (2019). It takes a village: Phagocytes play a central role in fungal immunity. Semin. Cell Dev. Biol..

[69] Jhingran A., Kasahara S., Shepardson K.M., Junecko B.A., Heung L.J., Kumasaka D.K., Knoblaugh S.E., Lin X., Kazmierczak B.I., Reinhart T.A. (2015). Compartment-specific and sequential role of MyD88 and CARD9 in chemokine induction and innate defense during respiratory fungal infection. PLoS Pathog..

[70] Lehmann R., Muller M.M., Klassert T.E., Driesch D., Stock M., Heinrich A., Conrad T., Moore C., Schier U.K., Guthke R. (2018). Differential regulation of the transcriptomic and secretomic landscape of sensor and effector functions of human airway epithelial cells. Mucosal Immunol..

[71] Heyl K.A., Klassert T.E., Heinrich A., Müller M.M., Klaile E., Dienemann H., Grünewald C., Bals R., Singer B.B., Slevogt H. (2014). Dectin-1 is expressed in human lung and mediates the proinflammatory immune response to nontypeable *Haemophilus influenzae*. mBio.

[72] Ordovas-Montanes J., Dwyer D.F., Nyquist S.K., Buchheit K.M., Vukovic M., Deb C., Wadsworth M.H., Hughes T.K., Kazer S.W., Yoshimoto E. (2018). Allergic inflammatory memory in human respiratory epithelial progenitor cells. Nature.

[73] Kawamoto Y., Morinaga Y., Kimura Y., Kaku N., Kosai K., Uno N., Hasegawa H., Yanagihara K. (2017). TNF-alpha inhibits the growth of *Legionella pneumophila* in airway epithelial cells by inducing apoptosis. J. Infect. Chemother..

[74] Chen F., Zhang C., Jia X., Wang S., Wang J., Chen Y., Zhao J., Tian S., Han X., Han L. (2015). Transcriptome profiles of human lung epithelial cells A549 interacting with *Aspergillus fumigatus* by RNA-seq. PLoS ONE.

[75] Oguma T., Asano K., Tomomatsu K., Kodama M., Fukunaga K., Shiomi T., Ohmori N., Ueda S., Takihara T., Shiraishi Y. (2011). Induction of mucin and MUC5AC expression by the protease activity of *Aspergillus fumigatus* in airway epithelial cells. J. Immunol..

[76] Feldman M.B., Dutko R.A., Wood M.A., Ward R.A., Leung H.M., Snow R.F., De La Flor D.J., Yonker L.M., Reedy J.L., Tearney G.J. (2020). *Aspergillus fumigatus* cell wall promotes apical airway epithelial recruitment of human neutrophils. Infect. Immun..

[77] Feldman M.B., Wood M., Lapey A., Mou H. (2019). SMAD signaling restricts mucous cell differentiation in human airway epithelium. Am. J. Respir. Cell Mol. Biol..

[78] Shivaraju M., Chitta U.K., Grange R.M.H., Jain I.H., Capen D., Liao L., Xu J., Ichinose F., Zapol W.M., Mootha V.K. (2021). Airway stem cells sense hypoxia and differentiate into protective solitary neuroendocrine cells. Science.

[79] Eenjes E., Mertens T.C.J., Buscop-van Kempen M.J., van Wijck Y., Taube C., Rottier R.J., Hiemstra P.S. (2018). A novel method for expansion and differentiation of mouse tracheal epithelial cells in culture. Sci. Rep..

[80] Yonker L.M., Pazos M.A., Lanter B.B., Mou H., Chu K.K., Eaton A.D., Bonventre J.V., Tearney G.J., Rajagopal J., Hurley B.P. (2017). Neutrophil-derived cytosolic PLA2α contributes to bacterial-induced neutrophil transepithelial migration. J. Immunol..

[81] Cheng D.S., Han W., Chen S.M., Sherrill T.P., Chont M., Park G.Y., Sheller J.R., Polosukhin V.V., Christman J.W., Yull F.E. (2007). Airway epithelium controls lung inflammation and injury through the NF-kappa B pathway. J. Immunol..

[82] Hernandez-Santos N., Wiesner D.L., Fites J.S., McDermott A.J., Warner T., Wuthrich M., Klein B.S. (2019). Lung Epithelial Cells Coordinate Innate Lymphocytes and Immunity against Pulmonary Fungal Infection. Cell Host Microbe.

[83] Wiesner D.L., Merkhofer R.M., Ober C., Kujoth G.C., Niu M., Keller N.P., Gern J.E., Brockman-Schneider R.A., Evans M.D., Jackson D.J. (2020). Club cell TRPV4 serves as a damage sensor driving lung allergic inflammation. Cell Host Microbe.

[84] Valque H., Gouyer V., Duez C., Leboeuf C., Marquillies P., Le Bert M., Plet S., Ryffel B., Janin A., Gottrand F. (2019). Muc5b-deficient mice develop early histological lung abnormalities. Biol. Open.

[85] Evans C.M., Raclawska D.S., Ttofali F., Liptzin D.R., Fletcher A.A., Harper D.N., McGing M.A., McElwee M.M., Williams O.W., Sanchez E. (2015). The polymeric mucin Muc5ac is required for allergic airway hyperreactivity. Nat. Commun..

[86] Ehre C., Worthington E.N., Liesman R.M., Grubb B.R., Barbier D., O’Neal W.K., Sallenave J.-M., Pickles R.J., Boucher R.C. (2012). Overexpressing mouse model demonstrates the protective role of Muc5ac in the lungs. Proc. Natl. Acad. Sci. USA.

[87] Sui P., Wiesner D.L., Xu J., Zhang Y., Lee J., Van Dyken S., Lashua A., Yu C., Klein B.S., Locksley R.M. (2018). Pulmonary neuroendocrine cells amplify allergic asthma responses. Science.

[88] Bays D., Thompson G.R., Reef S., Snyder L., Friefeld A., Huppert M., Salkin D., Wilson M., Galgiani J.N. (2020). Natural History of Disseminated Coccidioidomycosis: Examination of the Veterans Affairs–Armed Forces Database. Clin. Infect. Dis..

[89] Vinh D.C., Masannat F., Dzioba R.B., Galgiani J.N., Holland S.M. (2009). Refractory disseminated coccidioidomycosis and mycobacteriosis in interferon-gamma receptor 1 deficiency. Clin. Infect. Dis..

[90] Vinh D.C., Schwartz B., Hsu A.P., Miranda D.J., Valdez P.A., Fink D., Lau K.P., Long-Priel D., Kuhns D.B., Uzel G. (2011). Interleukin-12 receptor β1 deficiency predisposing to disseminated coccidioidomycosis. Clin. Infect. Dis..

[91] Odio C.D., Milligan K.L., McGowan K., Rudman Spergel A.K., Bishop R., Boris L., Urban A., Welch P., Heller T., Kleiner D. (2015). Endemic mycoses in patients with STAT3-mutated hyper-IgE (Job) syndrome. J. Allergy Clin. Immunol..

[92] Sade-Feldman M., Yizhak K., Bjorgaard S.L., Ray J.P., de Boer C.G., Jenkins R.W., Lieb D.J., Chen J.H., Frederick D.T., Barzily-Rokni M. (2018). Defining T Cell States Associated with Response to Checkpoint Immunotherapy in Melanoma. Cell.

[93] Delorey T.M., Ziegler C.G.K., Heimberg G., Normand R., Yang Y., Segerstolpe A., Abbondanza D., Fleming S.J., Subramanian A., Montoro D.T. (2021). A single-cell and spatial atlas of autopsy tissues reveals pathology and cellular targets of SARS-CoV-2. bioRxiv.

[94] Chapuy L., Bsat M., Rubio M., Harvey F., Motta V., Schwenter F., Wassef R., Richard C., Deslandres C., Nguyen B.N. (2020). Transcriptomic Analysis and High-dimensional Phenotypic Mapping of Mononuclear Phagocytes in Mesenteric Lymph Nodes Reveal Differences Between Ulcerative Colitis and Crohn’s Disease. J. Crohn’s Colitis.

[95] Regev A., Teichmann S.A., Lander E.S., Amit I., Benoist C., Birney E., Bodenmiller B., Campbell P., Carninci P., Clatworthy M. (2017). The Human Cell Atlas. eLife.

[96] Schiller H.B., Montoro D.T., Simon L.M., Rawlins E.L., Meyer K.B., Strunz M., Vieira Braga F.A., Timens W., Koppelman G.H., Budinger G.R.S. (2019). The Human Lung Cell Atlas: A High-Resolution Reference Map of the Human Lung in Health and Disease. Am. J. Respir. Cell Mol. Biol..

[97] Ozsolak F., Milos P.M. (2011). RNA sequencing: Advances, challenges and opportunities. Nat. Rev. Genet..

[98] Picelli S., Björklund Å.K., Faridani O.R., Sagasser S., Winberg G., Sandberg R. (2013). Smart-seq2 for sensitive full-length transcriptome profiling in single cells. Nat. Methods.

[99] Hashimshony T., Senderovich N., Avital G., Klochendler A., de Leeuw Y., Anavy L., Gennert D., Li S., Livak K.J., Rozenblatt-Rosen O. (2016). CEL-Seq2: Sensitive highly-multiplexed single-cell RNA-Seq. Genome Biol..

[100] Klein A.M., Mazutis L., Akartuna I., Tallapragada N., Veres A., Li V., Peshkin L., Weitz D.A., Kirschner M.W. (2015). Droplet barcoding for single-cell transcriptomics applied to embryonic stem cells. Cell.

[101] Macosko E.Z., Basu A., Satija R., Nemesh J., Shekhar K., Goldman M., Tirosh I., Bialas A.R., Kamitaki N., Martersteck E.M. (2015). Highly parallel genome-wide expression profiling of individual cells using nanoliter droplets. Cell.

[102] Zheng G.X., Terry J.M., Belgrader P., Ryvkin P., Bent Z.W., Wilson R., Ziraldo S.B., Wheeler T.D., McDermott G.P., Zhu J. (2017). Massively parallel digital transcriptional profiling of single cells. Nat. Commun..

[103] Gierahn T.M., Wadsworth M.H., Hughes T.K., Bryson B.D., Butler A., Satija R., Fortune S., Love J.C., Shalek A.K. (2017). Seq-Well: Portable, low-cost RNA sequencing of single cells at high throughput. Nat. Methods.

[104] Cao J., Packer J.S., Ramani V., Cusanovich D.A., Huynh C., Daza R., Qiu X., Lee C., Furlan S.N., Steemers F.J. (2017). Comprehensive single-cell transcriptional profiling of a multicellular organism. Science.

[105] Ding J., Adiconis X., Simmons S.K., Kowalczyk M.S., Hession C.C., Marjanovic N.D., Hughes T.K., Wadsworth M.H., Burks T., Nguyen L.T. (2020). Systematic comparison of single-cell and single-nucleus RNA-sequencing methods. Nat. Biotechnol..

[106] Papalexi E., Satija R. (2018). Single-cell RNA sequencing to explore immune cell heterogeneity. Nat. Rev. Immunol..

[107] Mereu E., Lafzi A., Moutinho C., Ziegenhain C., McCarthy D.J., Álvarez-Varela A., Batlle E., Sagar, Grün D., Lau J.K. (2020). Benchmarking single-cell RNA-sequencing protocols for cell atlas projects. Nat. Biotechnol..

[108] Stoeckius M., Zheng S., Houck-Loomis B., Hao S., Yeung B.Z., Mauck W.M., Smibert P., Satija R. (2018). Cell hashing with barcoded antibodies enables multiplexing and doublet detection for single cell genomics. Genome Biol..

[109] Stoeckius M., Hafemeister C., Stephenson W., Houck-Loomis B., Chattopadhyay P.K., Swerdlow H., Satija R., Smibert P. (2017). Simultaneous epitope and transcriptome measurement in single cells. Nat. Methods.

[110] Li B., Gould J., Yang Y., Sarkizova S., Tabaka M., Ashenberg O., Rosen Y., Slyper M., Kowalczyk M.S., Villani A.C. (2020). Cumulus provides cloud-based data analysis for large-scale single-cell and single-nucleus RNA-seq. Nat. Methods.

[111] Gaublomme J.T., Li B., McCabe C., Knecht A., Yang Y., Drokhlyansky E., Van Wittenberghe N., Waldman J., Dionne D., Nguyen L. (2019). Nuclei multiplexing with barcoded antibodies for single-nucleus genomics. Nat. Commun..

[112] Dixit A., Parnas O., Li B., Chen J., Fulco C.P., Jerby-Arnon L., Marjanovic N.D., Dionne D., Burks T., Raychowdhury R. (2016). Perturb-seq: Dissecting molecular circuits with scalable single-cell RNA profiling of pooled genetic screens. Cell.

[113] Adamson B., Norman T.M., Jost M., Cho M.Y., Nuñez J.K., Chen Y., Villalta J.E., Gilbert L.A., Horlbeck M.A., Hein M.Y. (2016). A multiplexed single-cell CRISPR screening platform enables sytstematic dissection of the unfolded protein response. Cell.

[114] Jaitin D.A., Weiner A., Yofe I., Lara-Astiaso D., Keren-Shaul H., David E., Salame T.M., Tanay A., van Oudenaarden A., Amit I. (2016). Dissecting immune circuits by linking CRISPR-pooled screens with single-cell RNA-seq. Cell.

[115] Datlinger P., Rendeiro A.F., Schmidl C., Krausgruber T., Traxler P., Klughammer J., Schuster L.C., Kuchler A., Alpar D., Bock C. (2017). Pooled CRISPR screening with single-cell transcriptome readout. Nat. Methods.

[116] Gasperini M., Hill A.J., McFaline-Figueroa J.L., Martin B., Kim S., Zhang M.D., Jackson D., Leith A., Schreiber J., Noble W.S. (2019). A genome-wide framework for mapping gene regulation via cellular genetic screens. Cell.

[117] Villani A.C., Satija R., Reynolds G., Sarkizova S., Shekhar K., Fletcher J., Griesbeck M., Butler A., Zheng S., Lazo S. (2017). Single-cell RNA-seq reveals new types of human blood dendritic cells, monocytes, and progenitors. Science.

[118] Björklund Å.K., Forkel M., Picelli S., Konya V., Theorell J., Friberg D., Sandberg R., Mjösberg J. (2016). The heterogeneity of human CD127(+) innate lymphoid cells revealed by single-cell RNA sequencing. Nat. Immunol..

[119] Gaublomme J.T., Yosef N., Lee Y., Gertner R.S., Yang L.V., Wu C., Pandolfi P.P., Mak T., Satija R., Shalek A.K. (2015). Single-cell genomics unveils critical regulators of Th17 cell pathogenicity. Cell.

[120] De Vries D.H., Matzaraki V., Bakker O.B., Brugge H., Westra H.J., Netea M.G., Franke L., Kumar V., van der Wijst M.G.P. (2020). Integrating GWAS with bulk and single-cell RNA-sequencing reveals a role for LY86 in the anti-*Candida* host response. PLoS Pathog..

[121] Pechkovsky D.V., Goldmann T., Vollmer E., Müller-Quernheim J., Zissel G. (2006). Interleukin-18 expression by alveolar epithelial cells type II in tuberculosis and sarcoidosis. FEMS Immunol. Med. Microbiol..

[122] Aoki K., Matsumoto S., Hirayama Y., Wada T., Ozeki Y., Niki M., Domenech P., Umemori K., Yamamoto S., Mineda A. (2004). Extracellular mycobacterial DNA-binding protein 1 participates in mycobacterium-lung epithelial cell interaction through hyaluronic acid. J. Biol. Chem..

[123] Volkman H.E., Pozos T.C., Zheng J., Davis J.M., Rawls J.F., Ramakrishnan L. (2010). Tuberculous granuloma induction via interaction of a bacterial secreted protein with host epithelium. Science.

[124] Petrini B., Sköld C.M., Bronner U., Elmberger G. (2003). Coccidioidomycosis mimicking lung cancer. Respir. Int. Rev. Thorac. Dis..

[125] Azar M.M., Muse V.V., Villalba J.A., Turbett S.E. (2020). Case 2-2020: A 64-year-old man with fever and respiratory failure. N. Engl. J. Med..

[126] Bolaji O.M., Zainudin N.I., Snape S., Saini G., Baskaran V. (2021). Images of the month: The conundrum of chronic coccidioidomycosis. Clin. Med..

[127] Asp M., Bergenstråhle J., Lundeberg J. (2020). Spatially resolved transcriptomes-next generation tools for tissue exploration. Bioessays.

[128] Femino A.M., Fay F.S., Fogarty K., Singer R.H. (1998). Visualization of single RNA transcripts *In Situ*. Science.

[129] Raj A., van den Bogaard P., Rifkin S.A., van Oudenaarden A., Tyagi S. (2008). Imaging individual mRNA molecules using multiple singly labeled probes. Nat. Methods.

[130] Wang F., Flanagan J., Su N., Wang L.C., Bui S., Nielson A., Wu X., Vo H.T., Ma X.J., Luo Y. (2012). RNAscope: A novel in situ RNA analysis platform for formalin-fixed, paraffin-embedded tissues. J. Mol. Diagn. JMD.

[131] Annaratone L., Simonetti M., Wernersson E., Marchiò C., Garnerone S., Scalzo M.S., Bienko M., Chiarle R., Sapino A., Crosetto N. (2017). Quantification of HER2 and estrogen receptor heterogeneity in breast cancer by single-molecule RNA fluorescence in situ hybridization. Oncotarget.

[132] Ke R., Mignardi M., Pacureanu A., Svedlund J., Botling J., Wählby C., Nilsson M. (2013). In situ sequencing for RNA analysis in preserved tissue and cells. Nat. Methods.

[133] Lee J.H., Daugharthy E.R., Scheiman J., Kalhor R., Ferrante T.C., Terry R., Turczyk B.M., Yang J.L., Lee H.S., Aach J. (2015). Fluorescent in situ sequencing (FISSEQ) of RNA for gene expression profiling in intact cells and tissues. Nat. Protoc..

[134] Liu S., Punthambaker S., Iyer E.P.R., Ferrante T., Goodwin D., Fürth D., Pawlowski A.C., Jindal K., Tam J.M., Mifflin L. (2021). Barcoded oligonucleotides ligated on RNA amplified for multiplexed and parallel in situ analyses. Nucleic Acids Res..

[135] Zollinger D.R., Lingle S.E., Sorg K., Beechem J.M., Merritt C.R. (2020). GeoMx™ RNA assay: High multiplex, digital, spatial analysis of RNA in FFPE tissue. Methods Mol. Biol..

